# Flow Field Analysis Inside and at the Outlet of the Abrasive Head

**DOI:** 10.3390/ma14143919

**Published:** 2021-07-14

**Authors:** Zdenek Riha, Michal Zelenak, Kamil Soucek, Antonin Hlavacek

**Affiliations:** 1 Department of Material Disintegration, Institute of Geonics of the Czech Academy of Sciences, 60200 Brno, Czech Republic; zdenek.riha@ugn.cas.cz; 2 Department of Material Disintegration, Institute of Geonics of the Czech Academy of Sciences, 70800 Ostrava, Czech Republic; 3 Department of Geomechanics and Mining Research, Institute of Geonics of the Czech Academy of Sciences, 70800 Ostrava, Czech Republic; kamil.soucek@ugn.cas.cz; 4 Department of Biological Instrumentation, Institute of Analytical Chemistry of the Czech Academy of Sciences, 60200 Brno, Czech Republic; hlavacek@iach.cz

**Keywords:** abrasive injection cutting head, high-speed abrasive water jet, numerical flow modelling, particle tracking velocimetry, measurement of abrasive particle velocities

## Abstract

This paper focuses on the investigation of a multiphase flow of water, air, and abrasive particles inside and at the outlet of the abrasive head with the help of computational fluid dynamics calculations and measurements. A standard abrasive head with a water nozzle hole diameter of 0.33 mm (0.013”) and an abrasive nozzle cylindrical hole diameter of 1.02 mm (0.04”) were used for numerical modelling and practical testing. The computed tomography provided an exact 3D geometrical model of the cutting head that was used for the creation of the model. Velocity fields of abrasive particles at the outlet of the abrasive head were measured and analysed using particle tracking velocimetry and, consequently, compared with the calculated results. The calculation model took the distribution of the abrasive particle diameters with the help of the Rosin-Rammler function in intervals of diameters from 150 to 400 mm. In the present study, four levels of water pressure (105, 194, 302, 406 MPa) and four levels of abrasive mass flow rate (100, 200, 300, 400 kg/min) were combined. The values of water pressures and hydraulic powers measured at the abrasive head inlet were used as boundary conditions for numerical modelling. The hydraulic characteristics of the water jet were created from the measured and calculated data. The calculated pressure distribution in the cylindrical part of the abrasive nozzle was compared with studies by other authors. The details of the experiments and calculations are presented in this paper.

## 1. Introduction

Abrasive water jet (AWJ) is a progressive technology due to its various advantages. It is currently applied in the manufacturing industry, especially for processing difficult-to-machine materials such as ceramics, composites, and alloys [1,2]. AWJ is suitable for different technological operations such as machining, cutting, turning, cleaning, engraving, and polishing [3]. In addition to these technological operations, AWJs can also be used to simulate abrasive erosion on material surfaces loaded by fast-flowing fluid. Various methods based on accelerated mechanical modelling of the interaction of water flows with the surfaces of various materials have been investigated in [4]. The method using AWJs has been successfully applied for the determination of the erosion resistance of concretes treated with a solution of modified lithium silicates [5]. The influence of abrasive properties on the simulation of the abrasive erosion of cementitious composites was then analysed in [5]. The great potential of AWJ and its unique cutting ability were demonstrated during turning of difficult-cut materials such as Incoloy alloy 925 in [6] and cutting of stainless steel published in [7].

The disintegration phenomenon is based on the transmission of high-speed water jet energy into a small area of material. Material is then eroded by the collision of the mixture of water, air, and abrasive particles with the material’s surface. Due to the absence of a heat-affected zone in the area of interaction of the jet with a material, some negative effects are eliminated (for example, changes in the local material hardness, strength, or creep [8]).

**Figure 1:**
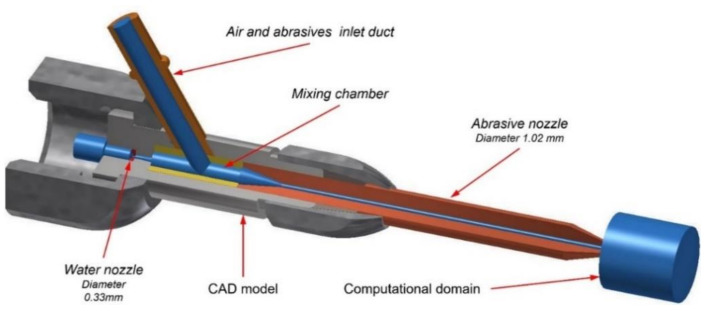
CAD model and computational domain of the abrasive head.

The process of AWJ generation in an injection abrasive head was described by Momber et al. [1]. In general, a high-speed water jet is generated by high-pressure liquid passing through a water nozzle where the pressure energy is transformed into kinetic energy and dissipation energy. Water jets can reach a velocity of more than 850 m/s. As the movement of the high-speed water jet along the axis of the cutting tool generates low pressure inside a mixing chamber, the mixture of abrasive particles and air is automatically sucked into the mixing chamber through a side hole connected to the inlet duct. Subsequently, abrasive particles and air are accelerated due to the kinetic energy of the high-speed water jet flowing into the mixing chamber and abrasive nozzle. The generated abrasive water jet can thus be applied for cutting of any material [9]. The basic principles of AWJ generation and its applications are summarised in the publication by Liu et al. [2]. Figure 1 displays the CAD model and computational domain (blue colour) of the abrasive head used in the current study for numerical modelling and experimental testing.

In general, it is difficult to measure the process of AWJ generation and interaction with material by direct observation methods, since the experimental techniques are continuously developing. Research activities focusing on the AWJ processing of materials based on numerical simulations and direct observations can be divided into four categories: impact mechanics of abrasive particles during material interaction, numerical simulation of jet interaction with a solid surface, numerical simulation of the AWJ flow, and direct AWJ measurement methods. 

### 1.1. Background of AWJ Calculations

Numerical simulations are an effective tool for describing the AWJ generation process and subsequent interaction with a material. In particular, computational fluid dynamics (CFD) is a suitable numerical method for studying the AWJ flow. Liu et al. [10,11] developed CFD models for three-phase flow at the nozzle outlet based on 2D axisymmetric geometry, significantly simplifying the mixing chamber’s inner shape. Calculation of continuous phases (water, air) was realised in the first step, and the movement of abrasive particles was solved in the following step. The two-dimensional axisymmetric model was simple and quick, enabling the calculation of the distribution of water jet velocity along the domain axis with reasonable accuracy and the prediction of particle velocity inside the water jet [9]. Thongkaew and Wang [12] recently published a new numerical study focused on a model of the AWJ’s interaction with a solid surface in 2D geometry. The axisymmetric computational domain consisted of the area of the high-speed water jet with abrasives, air, and a rigid surface target. Calculated data of an inaccurate model were adjusted and verified using experimental methods of particle velocity measurement at the outlet of the abrasive nozzle, i.e., the PTV and laser-induced fluorescence (LIF). However, compared to the real geometry of the abrasive head, the computational domain was too simplified and did not provide any information about fluid flow in the inner space of the cutting tool (i.e., abrasive head).

With the development of computer software and with better availability of high-performance hardware, multiphase flow calculations have been conducted in 3D geometry. The calculations of mixture flow in 3D geometry of an abrasive head is described by Prisco et al. [13]. The two-phase flow was modelled using the sophisticated Euler-Euler approach that enabled the description of a wider range of individual flow phase interactions. The calculated results were compared with the reference model of Yanada et al. [14]. The results showed possible distributions of pressures, velocities, and densities in all parts of the abrasive head, unfortunately without the presence of abrasive particles. Moreover, the exact geometry of the water nozzle was not included in the computational domain. Qiang et al. [15] recently published a comprehensive numerical model of a three-phase flow in the abrasive cutting head. Several case studies were described in the paper including a change in the geometry’s arrangement of the supply duct in the sense of inclination to the tool axis and different positions of abrasives in the inlet’s profile. The paper also presented the results of the velocity distribution inside the abrasive head and at its outlet as well as the erosive wear of the inner parts. The calculated results of the air volume flow rate and particle velocities showed good agreement with the measured values. However, the model included only a one-way coupling discrete particle method (DPM) with continual phases (i.e., water and air), and the model did not describe the exact geometry of the water nozzle. All the mentioned studies were solved as time-independent tasks using two-equation Reynolds-averaged Navier-Stokes (RANS) turbulent models. The results of the CFD modelling inside the abrasive cutting head were not verified by practical tests. 

Several authors have focused on the development of an analytical model of flow in the cutting head. The model designed by Tazibt et al. [16] consisted of the analytical resolution of the differential and nonlinear equation of particle motion within a high-speed water jet flowing in an abrasive nozzle. However, the model did not consider the effect of particle size distribution, and its validation was affected by limited access to experimental data. Momber [17] presented a model predicting abrasive velocities based on impact force measurement on a workpiece. Development of experimental methods enhanced new numerical simulations and verification of local conditions. Narayanan et al. [18] designed a phenomenological model of three-phase flow inside an abrasive water jet that was subsequently verified by experiments. The aim of their calculations was a complex study of flow characteristics during AWJ generation with the main focus on the determination of abrasive particle velocities at the focusing tube outlet. In this case, the abrasive particle size distribution and the effect of the breakage of particles on the energy flux were taken into consideration. The above-mentioned models focused on one part of the complex geometry of the abrasive head (i.e., abrasive nozzle) with a limited number of parameters (i.e., high-speed water jet velocity and abrasive particle velocity).

### 1.2. Background of Abrasive Particle Velocity Measurement

It is obvious that the outlet velocity and the mass volume of abrasive grains significantly influence the cutting ability of AWJs. Therefore, there has been a large effort to study abrasive particle velocity by direct observation methods. The first experimental studies describing the observation of abrasive particle velocities were published in the late 1990s. Only non-optical methods were applied. Swanson et al. [19] tested an inductive method using coils in combination with 10% magnetic tracer particles and standard abrasive grains. However, the measured particle velocities in this experiment reached the velocity of the pure water jet. Isobe et al. [20] used fast rotary discs enabling velocity calculation based on erosion dimensions in the disc. Li et al. [21] tested the estimation of high-speed water jet dynamics using force measurement. The authors used two Kistler piezoelectric transducers measuring three force components. The disadvantage was a low validation of the calculated velocities. A recent study published by Haghbin et al. [22] using a modified rotating dual-disc anemometer provided very good agreement with the measured results of the existing analytical models of abrasive particle velocity. 

AWJ observation was improved using laser Doppler velocimetry (LDV) and laser transit anemometers (LTA). LDV has been used in fluid research as a method for velocity flow investigation in a variety of applications. Neusen et al. [23,24] investigated the velocity of an AWJ and of a pure water jet (WJ). The presented studies described measurement of the pure water jet. However, the differentiation of water droplets and solid particles in the jet was very low due to the spatial resolution. Chen and Geskin [25] tested the LTA with a He-Ne laser. Two laser beams splitting from a single incoming laser were focused onto a small region at two focal points. An abrasive particle passing through the focal point of either of these split beams generated a scattering light that generated a signal. Particle velocity was calculated based on the known distance between two focal points and the time delay. Here, again, the disadvantage was the low spatial resolution.

Roth, Baltz, and Heiniger et al. [26] found a solution to improve the measurement accuracy of abrasive particle velocities using the LIF method combined with the planar PTV. A camera sensor captured a light signal emitted by fluorescent abrasive particles. As a source for particle illumination, a high-powered Nd:YAG laser was used. As a result, 2D vector maps of individually detected particles were created. Heiniger improved this method using a stereo system consisting of two CCD cameras [27] that provided 3D vector fields and particle distribution. This method is thus considered the best observation method. A crucial part of the successful measurements was the stability of the fluorescent coating on the particles’ surface under the test conditions presented in the conclusions published by the above-mentioned authors. Zelenak et al. [28] improved the coating procedure of fluorescent abrasive grains, which was successfully applied for observation of abrasive particle velocities in the abrasive suspension water jet, where the presence of fluorescent particles in a wet environment is required. The latest research published by Jerman et al. [29] introduced the application of a planar laser-induced fluorescence method for observation of ice particles in a high-speed water jet.

### 1.3. Background Summary

It is obvious from the previous investigations that flow modelling during AWJ generation and particle velocities at the nozzle outlet were studied. However, in many cases, the presented numerical studies were too simplified, and an inaccurate geometry of the computational domain of the abrasive head was applied. In addition, the models were solved with a uniform particle size distribution. The presented phenomenological numerical studies [16] supported by experiments were validated for laboratory conditions with water pressures up to 300 MPa. Investigations of CFD studies supported by experiments using pressures of up to 400 MPa and higher are still missing.

The aim of this study was to develop a sufficiently accurate and quick CFD model of the multiphase mixture flow inside a cutting head and at its outlet (Figure 1). For the purpose of the computational domain, an experimental water nozzle geometry was created based on direct computed tomography (CT) scanning. High-pressure flow sensors were used for hydraulic power measurement of the input water. PTV was used for the analysis of abrasive particle velocity fields at the outlet of an abrasive head. The computed CFD results of abrasive particle velocities were compared with a direct method of abrasive particle velocity observation. The size distribution of abrasive particles applied in the CFD computation corresponded with the real size of particles used during experimental testing. The methods used are described in more detail in the following sections.

## 2. Methods

A standard abrasive head with a water nozzle hole diameter of 0.33 mm (0.013”) and an abrasive nozzle cylindrical hole diameter of 1.02 mm (0.04”) were used for numerical modelling and practical testing. The calculations and experiments were performed with inlet water pressures of 105, 194, 302, and 406 MPa, and mass flow rates of 100, 200, 300, and 400 g/min.

### 2.1. Water Nozzle Geometrical Reconstruction Based on X-ray CT

**Figure 2:**
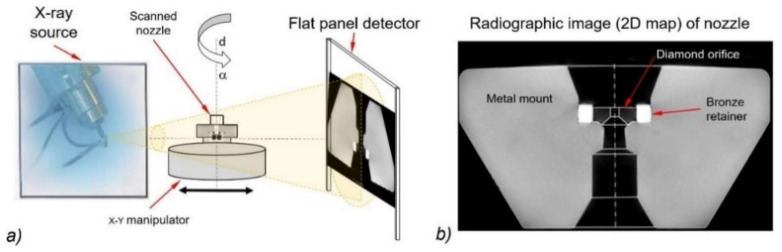
X-ray CT scheme: (**a**) projection of the water nozzle using the CT method; (**b**) 2D image of the water nozzle.

The exact geometry of the abrasive head’s computational domain is the first necessary condition for obtaining the correct calculation data in relation to the measured data. Computed tomography (CT) scanning was used to obtain the exact inner geometry of the water nozzle. The volumetric model of the water nozzle was acquired using a Nikon XT H225 CT scanner (Nikon Metrology Europe NV, Leuven, Belgium) to take 1000 2D radiographic images. A 3D standard triangle language (*.stl) model was created from 2D radiographic images using the VG Studio Max software (Volume Graphics GmbH, Heidelberg, Germany). The given 3D STL model of the water nozzle was used for the subsequent construction of the computational domain (Figure 1). A scheme of the CT projection is presented in Figure 2a. Figure 2b shows a 2D picture of the water nozzle extracted from the abrasive cutting head before experimental tests. 

### 2.2. CFD Geometry of Computational Mesh, Model Settings, and Calculations 

**Figure 3:**
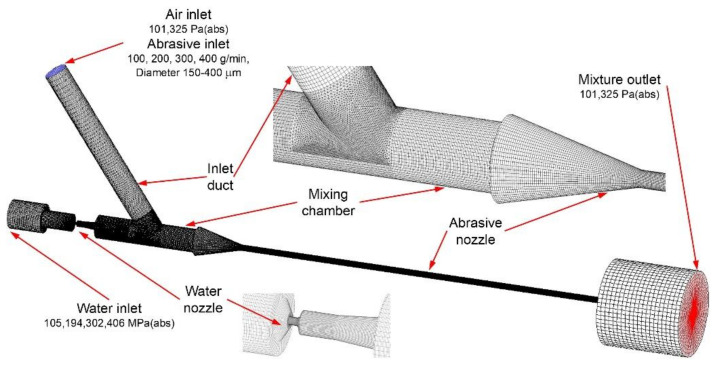
Mesh of the computational domain with boundary conditions.

The geometry of the computational domain was identical to the geometry of the abrasive head used in the experiment (Figure 3). The computational domain was meshed, consisting of the velocity, pressure, and density gradients. Mainly, hexahedral elements were used. Tetrahedral elements were applied in the transition area between the inlet duct and the mixing chamber. Pentahedral elements filled the rest of the domain space between the above-mentioned elements (Figure 3). The entire computational domain contained 2.3 million elements. This careful preparation of the computational domain geometry created good conditions for stable and convergent calculations and eliminated possible sources of errors and discrepancies between the calculated results and measured values.

For numerical calculation of the flow, the geometry of a multiphase mixture model [30] was selected. The mixture model was based on two basic equations, i.e., the Navier-Stokes equation and the mass equilibrium equation of continuum [30]. The Navier-Stokes equation describes the force equilibrium in a continuous environment. The general form of the equation can be expressed as:

(1) $$\frac{\partial }{\partial t}\left(\rho_{m}u_{m}{}_{i}\right)+\frac{\partial }{\partial x_{j}}\left(\rho_{m}u_{m}{}_{i}u_{m}{}_{j}\right)=-\frac{\partial p}{\partial x_{i}}+\rho_{m}g_{i}+F_{i}+\frac{\partial }{\partial x_{j}}\left[\mu_{m}\left(\frac{\partial u_{m}{}_{i}}{\partial x_{j}}+\frac{\partial u_{m}{}_{j}}{\partial x_{i}}-\frac{2}{3}\delta_{ij}\frac{\partial u_{m}{}_{l}}{\partial x_{l}}\right)\right]+\;\frac{\partial }{\partial x_{j}}\left(\mu_{m}{}_{t}\left(\frac{\partial u_{m}{}_{i}}{\partial x_{j}}+\frac{\partial u_{m}{}_{j}}{\partial x_{i}}\right)-\frac{2}{3}\left(\rho_{m}k_{m}+\mu_{m}{}_{t}\frac{\partial u_{m}{}_{l}}{\partial x_{l}}\right)\delta_{ij}\right)$$

where *t* is time, *ρ_m_* is the mixture density, *x_i_* is a position vector component in the *i*th direction, *x_j_* is a position vector component in the *j*th direction, *u_mi_* is a mixture velocity component in the *i*th direction, *u_mj_* is a mixture velocity component in the *j*th direction, *p* is the mixture pressure, *g_i_* is the gravity acceleration component in the *i*th direction, *F_i_* is the volume force component in the *i*th direction, *μ_m_* is the molecular viscosity of the mixture, *μ_mt_* is the turbulent viscosity of the mixture, *k_m_* is the turbulent kinetic energy of the mixture, and *δ_ij_* is the Kronecker delta. The mass equilibrium equation in continuum can be applied. The general form of the equation can be written as:

(2) $$\frac{\partial }{\partial t}\left(\rho_{m}\right)+\frac{\partial }{\partial x_{i}}\left(\rho_{m}u_{m}{}_{i}\right)=0$$

If the effect of slip velocity is neglected, the volume fraction *α_k_* of the secondary component of the mixture is calculated from the following equation:

(3) $$\frac{\partial }{\partial t}\left(\alpha_{k}\rho_{k}\right)+\frac{\partial }{\partial x_{i}}\left(\alpha_{k}\rho_{k}u_{m_{i}}\right)=\overset{n}{\underset{p=1}{\sum }}\left({\overset{•}{m}}_{pk}-{\overset{•}{m}}_{kp}\right)$$

Equation (3) is completed by the condition that the sum of all volume fractions in the given place equals 1:

(4) $$\overset{n}{\underset{k=1}{\sum }}\alpha_{k}=1$$

where *α_k_* is the volume fraction of the *k*-component of the mixture, *ρ_k_* is the density of the *k*-component of the mixture, *μ_k_* is the viscosity of the *k*-component of the mixture, *m_pk_* is the mass transfer from the phase *p* to *k*, and *m_kp_* is the mass transfer from the phase *k* to *p*. The two-equation RANS shear stress transport (SST) k-ω model [31] was used for the turbulent flow of water and air description. The general form of these equations can be written as:

(5) $$\frac{\partial }{\partial t}\left(\rho_{m}k_{m}\right)+\frac{\partial }{\partial x_{i}}\left(\rho_{m}k_{m}u_{m}{}_{i}\right)=\frac{\partial }{\partial x_{j}}\left(\Gamma_{k}\frac{\partial k_{m}}{\partial x_{j}}\right)+G_{k}-Y_{k}+S_{k},$$

(6) $$\frac{\partial }{\partial t}\left(\rho_{m}\omega_{m}\right)+\frac{\partial }{\partial x_{i}}\left(\rho_{m}\omega_{m}u_{m}{}_{i}\right)=\frac{\partial }{\partial x_{j}}\left(\Gamma_{\omega }\frac{\partial \omega_{m}}{\partial x_{j}}\right)+G_{\omega }-Y_{\omega }+D_{\omega }+S_{\omega },$$

where *ω_m_* is a specific ratio of the dissipation and turbulent energy, Γ*_k_* represents the effective diffusivity of *k_m_*, Γ*_ω_* represents the effective diffusivity of *ω_m_*, *G_k_* represents production of *k_m_*, *G_ω_* represents the generation of *ω_m_*, *Y_k_* represents the dissipation of *k_m_* due to the fact of turbulence, *Y_ω_* represents the dissipation of *ω_m_* due to the fact of turbulence, *D_ω_* is the cross-diffusion term, *S_k_* is the user-defined source term of *k_m_*, and *S_ω_* is the user-defined source term of *ω_m_*. The above-mentioned equations solved the fluid flow of water and air as continuum using the Euler approach. The flow of abrasive particles in a continuous environment of water and air was solved based on the Lagrange approach, using the DPM. The abrasive particle trajectories were computed individually at specified intervals during water and air calculations. This approach is suitable when particle-particle interactions are neglected. This requires that the solid particle phase occupies a low volume fraction, even though high mass loading is acceptable. At the same time, the particles should be sufficiently small with respect to the solved domain size [31]. The abrasive particle movement was solved by the following equation:

(7) $$\frac{\partial }{\partial t}\left(u_{p_{i}}\right)+\frac{\partial }{\partial x_{j}}\left(u_{pi}u_{pj}\right)=F_{D}\left(u_{mi}-u_{pi}\right)+\frac{g_{i}\left(\rho_{p}-\rho_{m}\right)}{\rho_{p}}+F_{pi}$$

(8) $$F_{D}=\frac{18\;\mu_{m}}{\rho_{p}d_{p}^{2}}\frac{C_{D}\;Re}{24},$$

where *u_pi_* is the component of particle velocity in the *i*th direction, *u_pj_* is the component of particle velocity in the *j*th direction, *F_D_* is the drag force, *ρ*_p_ is the particle density, *F_pi_* is the component of the additional force to the particle in the *i*th direction, *C_D_* is the drag coefficient, *Re* is the Reynolds number, and *d_p_* is the particle diameter. A two-way coupling method was used in the calculation of the particle interaction with the continuous phase [30]. The movement of abrasive particles affected the flow field shape of the continuous mixture given by water and air. Together with initial and boundary conditions (Figure 3), the above-mentioned equations formed a closed system that allowed for the calculation of an unambiguous solution of a turbulent flow field for the steady-state multiphase mixture in the solved domain. In particular, the effect of water compressibility was included in the calculation [32]. It was important to calculate the right shape of the velocity profiles at the outlet of the water nozzle, as the velocity profile results can differ significantly in incompressible and compressible fluid cases at pressures over 100 MPa. The water density was calculated using Equation (9):

(9) $$\rho =\frac{\rho_{ref}}{\left(1-\frac{K}{p-p_{ref}}\right)}$$

where *K* is the bulk modulus of water, *p* is the pressure, *ρ_ref_* is the reference density, and *p_ref_* is the reference pressure. DPM was selected for the description of solid particle movement in air and water. The mutual interaction between abrasive particles and continuous phases was set in the model in order to obtain a better accuracy of the calculated velocity distribution. It has been shown that the particle movement influences the velocity distribution, pressure, and density of the continuous phases. In the computational model, various diameters of abrasive particles were described using a special function called the Rosin-Rammler distribution function (10) [33]:

(10) $$Y_{d}=e^{-{\left(d/\bar{d}\right)}^{n}},$$

where *Y_d_* is the volume fraction of particles with a diameter greater than *d*, *d* is the particle diameter, $\bar{d}$ is the average diameter of particles, and *n* is the spreading exponent. Constant values for the average particle diameter, $\bar{d}$ = 281 μm, and the spreading exponent, *n* = 5.9, were calculated for the 150–400 μm range of abrasive particle diameters. Figure 4 shows a comparison of the calculated and measured grain size curves. Apparently, the curves are almost similar. According to the measured grain size curves, the distribution of abrasives used in the calculation simulates the near-real situation. Measurement of the abrasive particle distribution was performed using a Fritsch Nanosizer Analysette NanoTec 22 (FRITSCH GmbH, Oberstein, Germany).

Abrasive particles enter the computational domain through a supply duct (Figure 3). The particles are evenly distributed across the inlet section and enter the computational domain at an initial velocity lower than the velocity of the flowing air. The calculation of abrasive particle movement in the solved domain is retroactively influenced by the shape of the flow field of continuous phases (water, air). A process of air and abrasive particle acceleration decreases the velocity of the high-speed water jet. Long et al. [34] showed the importance of defining the shape of abrasive particles and their behaviour at the walls of the abrasive head. The calculation considers the energy loss in the tangential and normal directions due to the particle-wall contact. Specifications of restitution parameters of particles colliding with walls of the abrasive head are hard to find in any studies related to the topic; although, the setting of restitution parameters and particle sphericity directly influence the particle velocity magnitude at the outlet of the abrasive nozzle. However, Long et al. [35,36] evaluated data from experimental cases based on other physical phenomena than the multiphase fluid flow in the cutting tool. In the current study, the above-mentioned parameters were only estimated. The coefficient of restitution equalled 0.8 for both the tangential and the wall-normal direction of impacting particles. The particle sphericity was set as 0.65.

Standard values of under-relaxation factors were applied [30]. At the beginning of the calculations, the relaxation factor of the volume fraction was reduced to 0.1. After several hundred iterations, the mentioned value was gradually increased to 1. This way, it was allowed to reach of rapid convergence of the calculation. The recommended discretisation schemes were used: the SIMPLE scheme for the pressure velocity coupling, PRESTO scheme for the pressure, QUICK scheme for the volume fraction, and Second Order Upwind schemes for others [30]. The task was calculated on a workstation where a Windows Server 2008 HPC Edition 64-bit operating system (Microsoft Corporation, Redmond, Washington, DC, USA, accessed on 3 May 2009) was installed. The workstation was equipped with two six-core Intel Xeon X5690 processors and 48 GB of RAM (Intel corporation, Santa Clara, CA, USA). One task was calculated using eight cores of the mentioned processors. The calculation time was approximately 24 h. Special control of the calculation based on a gradual increase in under-relaxation parameters enabled the achievement of a very short calculation time. In total, sixteen tasks (combining various values of the feeding water pressure and the abrasive mass flow rate) were calculated.

### 2.3. High-Pressure Water Flow Monitoring in the Abrasive Head

**Figure 5:**
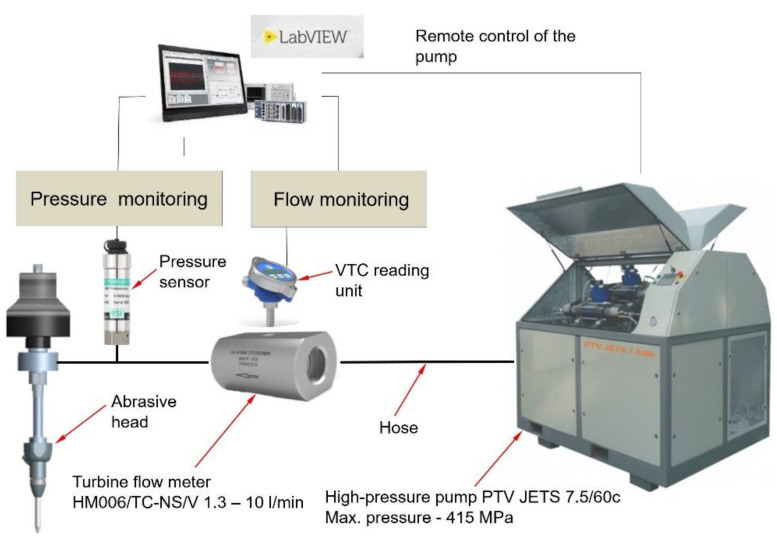
Experimental configuration of the flow and pressure monitoring.

The experimental assembly used for the high-pressure flow monitoring in the cutting head consisted of HM006/TC-NS/V turbine flow meter (KEM Küppers Elektromechanik GmbH, Karlsfeld, Germany) and an ESI GD pressure sensor (ESI Technology Ltd., Wrexham, UK) with a guaranteed accuracy of 0.1%. Experiments were performed using a high-pressure pump PTV Jets 7.5/60c (PTV, spol. s r.o., Praque, Czech Repubic). Hydraulic power for individual operating conditions was calculated based on the results of pressure and volume flow rate measurements. The measurement time for each test was 60 s with a sampling interval of 1 s to determine the average values of the measurement. The volume flow rate was determined in relation to the compressibility of water and changes in density at particular pressures. The configuration of the volume flow rate measurement in the abrasive head is graphically presented in Figure 5. The flow meter and pressure sensor were installed as close as possible to the inlet of the abrasive head for hydraulic loss elimination. During all tests, the water temperature was measured using a thermocouple in the pressure sensor and varied in the range of 24.0 ± 0.8 °C.

### 2.4. Measurement of Abrasive Particle Velocities at the Outlet of the Abrasive Head

Currently, the best method for particle observation and measurement of their velocities in a high-speed jet is the LIF method combined with the particle tracking velocimetry (PTV) technique, known as planar laser-induced fluorescence (PLIF) [29]. AWJ is a multiphase turbulent flow. Therefore, the LIF method for detection of abrasive particles within the water-air turbulent jet was applied. For our purpose, natural Australian garnet (GMA Garnet Group, Georges Terrace Perth, Western Australia) was coated with a fluorescent matter. Abrasive grains with dimensions of 150–400 µm were dispersed in a polyurethane-refined acrylic lacquer with Rhodamine B fluorescent dye (Thermo Fisher Scientific, Waltham, MA, USA). Subsequently, the particles were dried at 55 °C for 20 h. Next, free dye and dust on the particles’ surface were removed by water cleaning. Finally, the particles were dried again and separated using vibratory sieve shakers [28].

**Figure 6:**
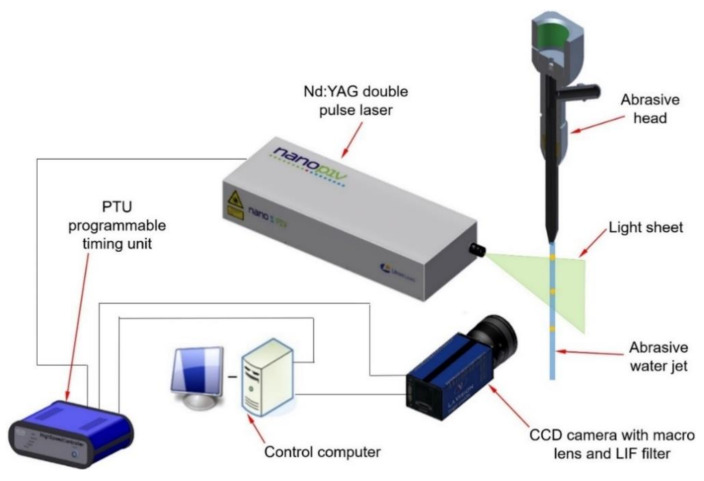
Planar PTV setup for the abrasive water jet visualisation.

The recording system (Figure 6) used for the visualisation and measurement of abrasive particle velocities within an AWJ consisted of a 14-bit CCD camera (LaVision GmbH, Goettingen, Germany) equipped with a special lens with a focal length of 60 mm, a LIF edge filter (LaVision GmbH, Goettingen, Germany), and a Nd:YAG double-pulse laser (LaVision GmbH, Goettingen, Germany). The laser generated thin light sheets (0.2 mm in the light sheet focus). The time period between the first and the second laser illumination varied in range up to 1 μs, depending on the AWJ’s operating parameters. The field of view (FOV) located at the outlet of the abrasive nozzle was 19.44 mm × 14.43 mm.

**Figure 7:**
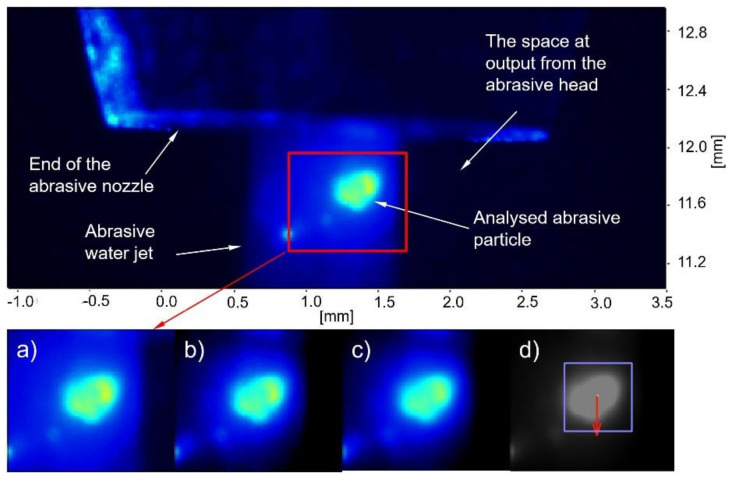
Image processing procedure: (**a**) experimental image; (**b**) background noise reduction; (**c**) averaging of pixel intensities; (**d**) tracking algorithm for abrasive particle velocity detection.

Under every tested pressure, a series of 100 double images were made. First, a subtracted offset algorithm was used to suppress the noise from the image background. The intensity of a particular pixel was averaged using intensities of neighbourhood pixels. At the end, velocities of abrasive particles were detected and evaluated by the tracking algorithm [29]. The image processing procedure is illustrated in Figure 7.

## 3. Results and Discussions

The results of the numerical modelling summarise the behaviour of a liquid flow (velocity, pressure, density, and particle movement). In addition, the direct measurements of the hydraulic characteristics under the tested pressures and observation of abrasive particle velocities at the abrasive nozzle outlet are presented. Numerical studies were consequently compared with the measurements. The domain of the abrasive head consisted of three parts. The first part was determined by flow conditions in the area around the water nozzle. The second part considered the space in the area of the mixing chamber and the abrasive nozzle. The third part focused on the space of the abrasive cutting head outlet.

### 3.1. Water Nozzle Space

The experimental results of the measured hydraulic power in the water nozzle are summarised in Table 1. The results show the dependence of the input feeding water pressure and the volume flow rate. Hydraulic power was counted as a simple product of feeding water pressure and volume flow rate.

**Figure 8:**
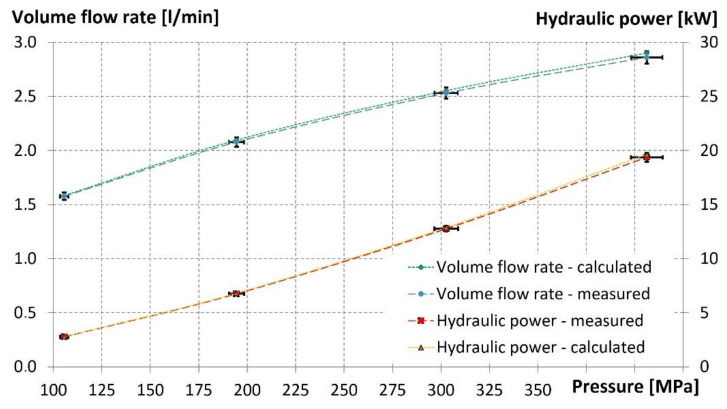
Measured and calculated volume flow rates and hydraulic power at the water nozzle inlet.

Measured and calculated values of the hydraulic power and of the volume flow rate are compared in relation to pressure levels in Figure 8.

The results obtained by numerical computation and by practical measurements reached very good agreement, with a margin of tolerance of ±2% due to the correct calculation settings using an appropriate internal geometry of the water nozzle determined by CT scanning.

**Figure 9:**
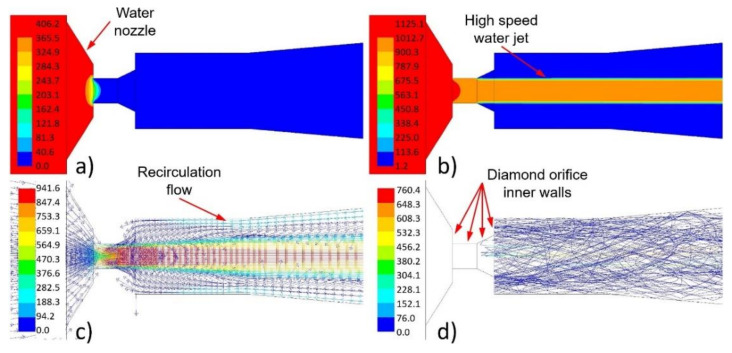
A graphical example of the calculated flow field shape around the water nozzle under the feeding pressure of 406 MPa: (**a**) distribution of the absolute pressure (MPa); (**b**) distribution of the density (kg/m^3^); (**c**) distribution of the velocity vector (m/s); (**d**) abrasive particle traces coloured by particle velocity magnitude (m/s)—abrasive particle mass flow rate of 100 g/min.

The water nozzle is a crucial part of the abrasive head. The orifice transforms the water pressure energy to the kinetic energy. This transformation is accompanied by a great increase in velocity, a large decrease in pressure, and a decrease in water density (Figure 9a–c). The increase in water feeding pressure is followed by an increase in the velocity of the high-speed water jet. The flow shape of the high-speed water jet is the same for all used feeding pressures. Geometric arrangement of the water nozzle and the mixing chamber enables recirculation of the air flow around the high-speed water jet (Figure 9c). This shape of flow fields caused abrasive particles to be sucked into to the orifice of the water jet. The abrasive particles thus hit the walls of the diamond orifice (Figure 9d).

Under other water feeding pressures (i.e., 106, 195, and 302 MPa), the flow field shapes were almost the same. The maximum values of pressure, velocity, and density were then proportionally lower.

### 3.2. Mixing Chamber and Abrasive Nozzle Space

**Figure 10:**
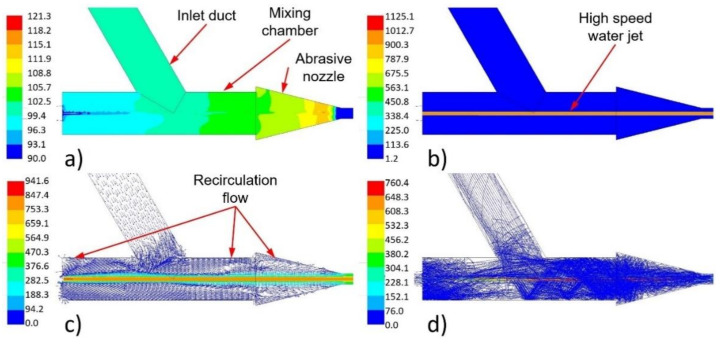
Calculated flow field in the mixing chamber and in the conical inlet part of the abrasive nozzle under the feeding pressure of 406 MPa: (**a**) distribution of the absolute pressure (kPa); (**b**) distribution of the density (kg/m^3^); (**c**) distribution of the velocity vector (m/s); (**d**) abrasive particle traces coloured by particle velocity magnitude (m/s)—abrasive particle mass flow rate of 100 g/min.

**Figure 11:**
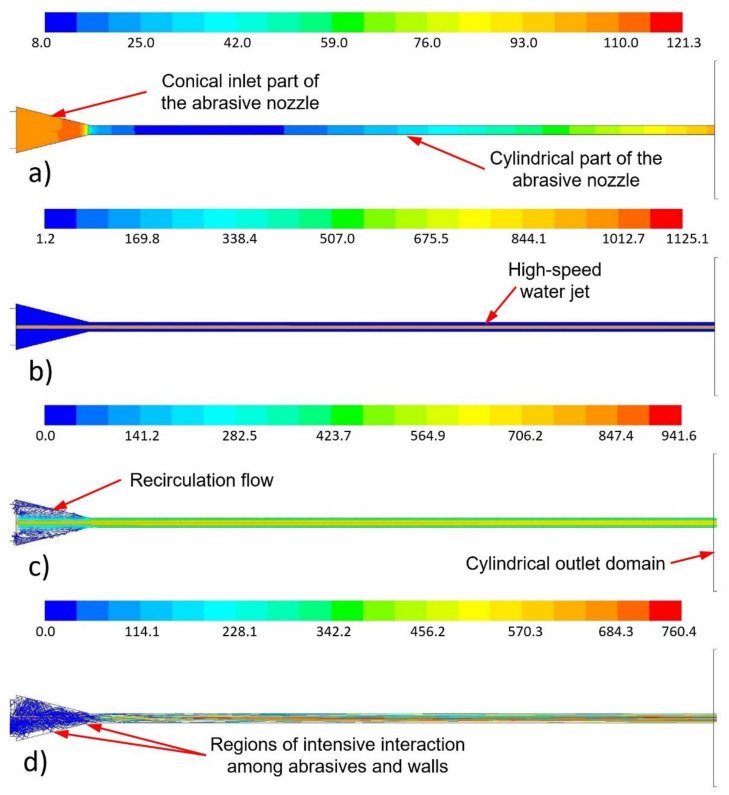
Calculated flow field in the abrasive nozzle under the feeding pressure of 406 MPa: (**a**) distribution of the pressure (kPa (abs)); (**b**) distribution of the density (kg/m^3^); (**c**) distribution of the velocity vector (m/s); (**d**) abrasive particle traces coloured by particle velocity magnitude (m/s)—abrasive particle mass flow rate of 100 g/min.

The numerical results show the behaviour of a liquid flow (i.e., pressure, velocity and density) and the abrasive particles’ movement in the space of the mixing chamber and the abrasive nozzle (Figure 10 and Figure 11).

**Figure 12:**
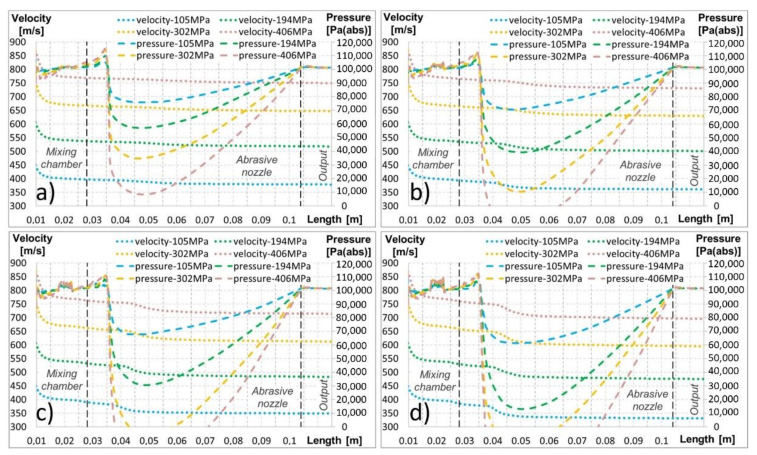
Calculated velocity and pressure distribution along the axis of the abrasive head, mixing chamber, focusing tube, and outlet: (**a**) abrasive mass flow rate of 100 g/min; (**b**) abrasive mass flow rate of 200 g/min; (**c**) abrasive mass flow rate of 300 g/min; (**d**) abrasive mass flow rate of 400 g/min.

The high-speed water jet flowed along the abrasive head axis through the mixing chamber and abrasive nozzle (Figure 10b and Figure 11b). The velocity decreased with the increasing distance from the water nozzle outlet due to the energy dissipation and energy transfer to air and abrasives (Figure 12). The high-speed water jet generated an ejector effect which caused the air and abrasive particles to be sucked into the mixing chamber through the inlet duct. The value of the pressure in the mixing chamber fluctuated slightly below the atmospheric pressure of 101.3 kPa (Figure 10a). A strong swirling flow of air was created around the high-speed water jet in the mixing chamber (Figure 10c). The movement of the abrasive particles was very messy there. The abrasives intensively flowed in the whole space of the mixing chamber. A number of abrasive particles hit the walls, and a few of them directly hit the high-speed water jet (Figure 10d). Particles bounced back, and some of them accelerated due to the high-speed water jet. The multiphase mixture continued into the conical inlet part of the abrasive nozzle. The flow field shape was similar to that in the mixing chamber. Again, the strong air swirl flow around the high-speed water jet and messy abrasive particle movement can be observed (Figure 10c,d and Figure 11c,d). The abrasive particles struck the walls with a high-velocity magnitude. Next, part of the abrasive nozzle was the cylindrical part. There, the flow field shape of the multiphase mixture was without strong recirculation zones and messy abrasive particle movement (Figure 11c,d). Air and the abrasives were further accelerated by the energy of the high-speed water jet as well as due to the narrow space of the cylindrical part of the abrasive nozzle (Figure 11d). The mentioned energy transport caused the velocity magnitude of the high-speed water jet to decrease, while the velocity magnitude of the air and the abrasives increased with the increasing length of the cylindrical part of the abrasive nozzle. The situation in this part of the calculated domain is illustrated in Figure 12. The solved domain consisted of the space of the mixing chamber, the abrasive nozzle, and the outlet cylinder. In the graphs, the interfaces of individual parts of the domain are marked by black vertical dashed lines (Figure 12). The velocity and pressure distribution along the abrasive head axis are presented for all the tested cases (Figure 12). It is obvious that the distribution of the curves is very similar for all tested pressures and abrasive mass flow rates. The curves shifted relative to each other according to given values of the water feeding pressure and the abrasive mass flow rate (Figure 12). The pressure increased over the length of the mixing chamber. In places where the abrasive particles directly hit the high-speed water jet, a local pressure increase occurred (for example, at the length of 0.02 m in Figure 12). The pressure rose gradually in the conical inlet part of the abrasive nozzle. The pressure culminated at the end of this part (at a length of 0.035 m, shown in Figure 12). In the cylindrical part of the abrasive nozzle, the pressure first rapidly decreased. Secondly, the pressure started to increase towards the end of the abrasive nozzle cylindrical part. Finally, the value of the atmospheric pressure was reached, which did not further change with the increasing length of the cylindrical part of the outlet domain (Figure 12). In this part of the abrasive nozzle, an increase in the feeding pressures and the abrasive mass flow rates caused a decrease in the pressure values (Figure 12).

Under higher levels of feeding water pressures (i.e., 302 and 406 MPa) and mass flow rates of abrasives (i.e., 200, 300, and 400 g/min), the minimum pressure in the abrasive nozzle could even fall below zero (Figure 12b–d). However, a negative value of the pressure was not physically possible. This was caused by a simplified description of the interaction between the continuous phase and the discrete phase in the numerical model.

Osman et al. studied the pressure dependences in a modified abrasive head [37]. Several measuring points in the inlet duct and in a special glass abrasive nozzle were performed, especially in its cylindrical part. The values of the pressure in these places were measured under various conditions of the high-speed water jet velocity or various values of the feeding water pressure. The measured pressure dependences in the cylindrical part of the abrasive nozzle were very close to the calculated ones. First, the pressure decreased. Then, with the increasing length of the abrasive nozzle, the pressure rose up to the atmospheric pressure at the end of the abrasive nozzle. The results of the measurement showed that an increase in the high-speed water jet’s velocity causes a decrease in the value of the minimum pressure in the abrasive nozzle [38] as observed in the calculation (see paragraphs above and Figure 12). The flow of the high-speed water jet inside and at the outlet of a glass abrasive nozzle was visualised as the next output of the paper published by Osman et al. [37]. The authors concluded that at the end of the abrasive nozzle, the high-speed jet expanded by filling its cross-sectional flow area. However, this conclusion did not correspond to the presented calculation results where the high-speed water jet kept almost the same diameter along the whole length of the computational domain without any significant expansion (Figure 11b).

### 3.3. Outlet Space of Abrasive Head

**Figure 13:**
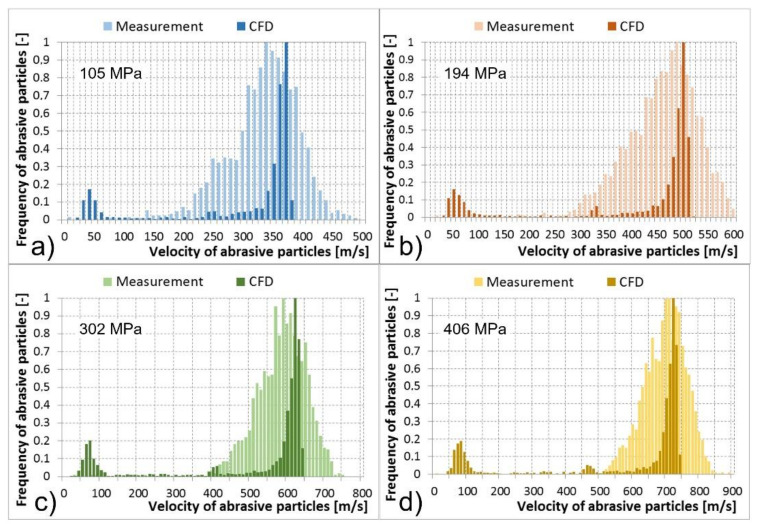
Abrasive particle velocities at the abrasive nozzle, measurement vs. computational models for given water pressure levels, and an abrasive particle mass flow rate of 100 g/min: (**a**) 105 MPa; (**b**) 194 MPa; (**c**) 302 MPa; (**d**) 406 MPa.

**Figure 14:**
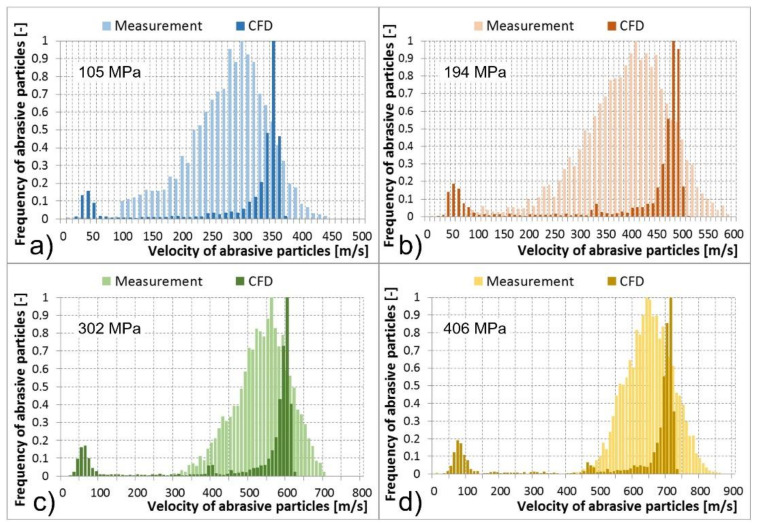
Abrasive particle velocities at the abrasive nozzle outlet, measurement vs. computational models for determined water pressure levels, and abrasive particle mass flow rate of 200 g/min: (**a**) 105 MPa; (**b**) 194 MPa; (**c**) 302 MPa; (**d**) 406 MPa.

**Figure 15:**
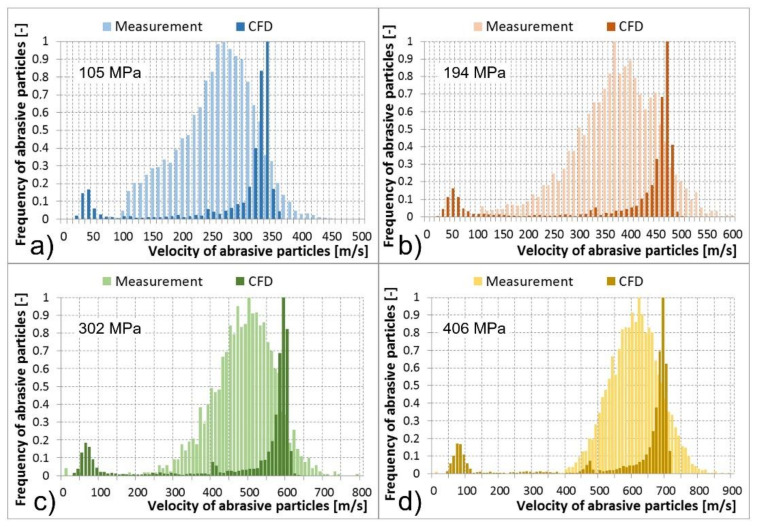
Abrasive particle velocities at the abrasive nozzle outlet, measurement vs. computational models for determined water pressure levels, and an abrasive particle mass flow rate of 300 g/min: (**a**) 105 MPa; (**b**) 194 MPa; (**c**) 302 MPa; (**d**) 406 MPa.

**Figure 16:**
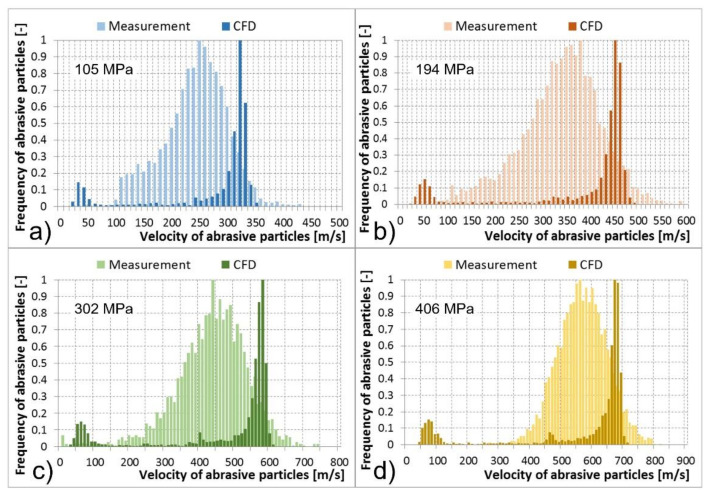
Abrasive particle velocities at the abrasive nozzle outlet, measurement vs. computational models for determined water pressure levels, and an abrasive particle mass flow rate of 400 g/min: (**a**) 105 MPa; (**b**) 194 MPa; (**c**) 302 MPa; (**d**) 406 MPa.

Multiphase mixture of the water, air, and abrasive particles exited the abrasive head, i.e., the abrasive nozzle. Results of abrasive particle velocities calculated using the CFD models and the PTV measurements are compared in the tables and histograms below (Table 2, Table 3, Table 4 and Table 5 and Figure 13, Figure 14, Figure 15 and Figure 16). The graphs illustrate the velocity distribution versus the frequency of abrasive particles. The calculated values are presented by dark colour and the measured values by light colour. In the numerical models, the particle velocities were evaluated at a distance of 0–14 mm behind the focusing tube outlet, which corresponded to the FOV of the camera used for optical measurement. The comparisons were performed under the determined conditions of the feeding water pressures and abrasive mass flow rates. The situation at the abrasive mass flow rate of 100 g/min is summarised in Table 2 and Figure 13. It is obvious that a higher level of the feeding water pressure causes acceleration of abrasive particles to higher velocities at the outlet of the abrasive nozzle. The same trend was valid for the average values of abrasive particle velocities presented in Table 2. The mentioned dependence between the feeding water pressure and abrasive particle velocities was true for both the measurements and CFD calculations. The measured values were distributed in wider intervals of velocities than the calculated values. Higher values of the abrasive particle velocities were reached during experimental testing compared to the calculations. The difference was approximately 100 m/s. This was valid for all tested and calculated feeding water pressures (Figure 13). In addition, both measured and calculated dependences reached one global maximum of the abrasive particle frequency in the area of higher velocities. In general, they were close to each other. The calculated global maxima were slightly higher; however, they were not more than 50 m/s in comparison to the measurement. The calculated dependences also showed one or two local maxima of frequency, mainly in the area of the lowest velocities as well as in the area of medium velocities (Figure 13). Therefore, the calculated dependences generated lower values of the average velocity of abrasive particles and higher values of the SD and SEM than the measured dependences (Table 2).

Cases with abrasive mass flow rates of 200, 300, and 400 g/min were very similar to the described case with an abrasive mass flow rate of 100 g/min. Nevertheless, an increase in the abrasive particle mass flow rates caused a greater difference in the location of global maxima of the calculated and measured frequencies of abrasive particles. The differences reached up to 100 m/s at abrasive mass flow rates of 200, 300, and 400 g/min; compared to the case of 100 g/min, where the difference was up to 50 m/s (Figure 13, Figure 14, Figure 15 and Figure 16). A shift in the global maxima was also visible considering the average velocities of abrasive particles (Table 2, Table 3, Table 4 and Table 5). For the case of 100 g/min, the calculated average velocity of abrasive particles was always smaller than the measured value (Table 2). However, at higher mass flow rates, the calculated average velocity of the abrasive particles began to become greater than the measured value at the determined feeding water pressures (Table 3, Table 4 and Table 5).

Generally, the increase in feeding water pressure raises the velocity of abrasive particles at the outlet of the abrasive head. On the contrary, the increase in the abrasive particle mass flow rate decreases the abrasive particle velocity at the outlet of the abrasive head. This description based on the measurement was also valid for the calculated data.

Nevertheless, several differences between the measured and calculated data at the outlet of the abrasive head can be observed. The first difference was based on the fact that the measured maxima of the abrasive particle velocities were higher than the calculated ones. The second difference showed that the measured global maxima were located lower than the calculated global maxima (Figure 13, Figure 14, Figure 15 and Figure 16). The high-speed water jet energy dissipation was higher in the numerical model. The selected Reynolds-averaged Navier-Stokes shear stress transport (RANS SST) k-ω turbulent model generated high-energy dissipation mainly between the water nozzle and the mixing chamber which caused lower values of the calculated maximum particle velocities at the outlet of the abrasive nozzle. The selected DPM calculated the movement of the abrasive particles separately, i.e., without the continuous phase calculations. Nevertheless, the discrete and continuous phases influenced each other. However, the mutual interactions only concerned the energy exchange. In reality, an abrasive particle struck the high-speed water jet and disturbed it. The jet thus flowed around the particle and accelerated it. The high-speed water jet disintegration due to the above-mentioned collision was not modelled. Therefore, the calculated and the measured dependencies differ by their shape (Figure 13, Figure 14, Figure 15 and Figure 16).

Significant local maxima of abrasive particle frequency can be observed in the area of the lowest velocities in all calculated cases (Figure 13, Figure 14, Figure 15 and Figure 16). The data obtained from measurements did not show this phenomenon. The mentioned calculated local maxima were caused by the density and velocity distribution of the continuous phases at the outlet of the abrasive head. High density and velocity values were observed in the middle of the outlet profile due to the high-speed water jet. Low-density and velocity values were distributed around the cylindrical wall of the abrasive nozzle due to the presence of air. The profile area with air contained a significant number of abrasive particles in the calculation. This caused the occurrence of local maxima in the graphs (Figure 13, Figure 14, Figure 15 and Figure 16).

During the abrasive head operation, solid particles collided with the inner walls of the mixing chamber and the abrasive nozzle. The collision of abrasive particles with the inner walls of the abrasive head was described by Kim et al. [38] and Hashish et al. [39]. They observed particle breakage and erosion wear of the abrasive head walls. Therefore, the grain size curve at the outlet had to be changed compared to the grain size curve at the inlet of the abrasive head. This phenomenon was not included in the numerical model, although the change in dimensions of abrasive particles can cause the above-described differences between measurements and calculations. The calculated values of the abrasive particle velocity at the outlet were consistent with the measured values considering the complexity of the mentioned phenomena occurring in the abrasive head.

## 4. Conclusions

A detailed description of a multiphase mixture flow inside and at the outlet of the abrasive head was presented in hitherto unpublished breadth. Sixteen settings for boundary conditions were compared within the measured and calculated data. Four levels of feeding water pressures (i.e., 105, 194, 302, and 406 MPa) were combined with four levels of abrasive mass flow rates (i.e., 100, 200, 300, and 400 g/min). Based on the performed calculations and measurements, the following was concluded:

- In the water nozzle, the calculated values of the volume flow rates were in very good conformity with the measured values under the determined feeding water pressures due to the exact geometry of the abrasive head that was included in the numerical model without simplifications. The exact geometry of the studied domain is thus a crucial part for creating and developing a numerical model which generates reasonable data;
- In the abrasive nozzle, a very good conformity of the pressure distribution in measurements and in calculations was observed. The shapes of dependences were similar with those published in other studies [38]. In the cylindrical part of the abrasive nozzle, the pressure decreased at first, then hit the minimum and, finally, grew up to the value of the atmospheric pressure. For both measurements and calculations, the increase in the high-speed water jet velocity caused a decrease in the minimum pressure;
- At the outlet of the abrasive head, a very good conformity of the abrasive particle velocity in measurements and calculations was reached due to the detailed description of abrasive particle diameters (from 150 to 400 μm) based on the function of the Rosin-Rammler particle size distribution used in calculations. For both measurements and calculations, the increase in the abrasive mass flow rate caused a decrease in the velocity of abrasive particles at the outlet of the abrasive head;
- A numerical model enabling a 3D multiphase steady-state turbulent visualisation of the flow in the space around the abrasive cutting head was developed. The numerical model provided stable and sufficiently accurate simulations in a short time without requiring extreme computing power.

The above-described experimental settings allowed for the obtention of important values of the cutting process generation that can be further used for validation of the calculated data. The experimental settings can be supplemented by measurements of pressure and velocity of air with abrasives at the inlet to the cutting head [40] as well as by the measurement of cutting effects [41] considering other parameters that also influence the accuracy of a cutting machine [42]. The combination of supplemented experimental settings with the presented numerical model creates suitable conditions for an effective optimisation of the abrasive head with respect to its lifetime and cutting efficiency.

## Figures and Tables

**Figure 4:**
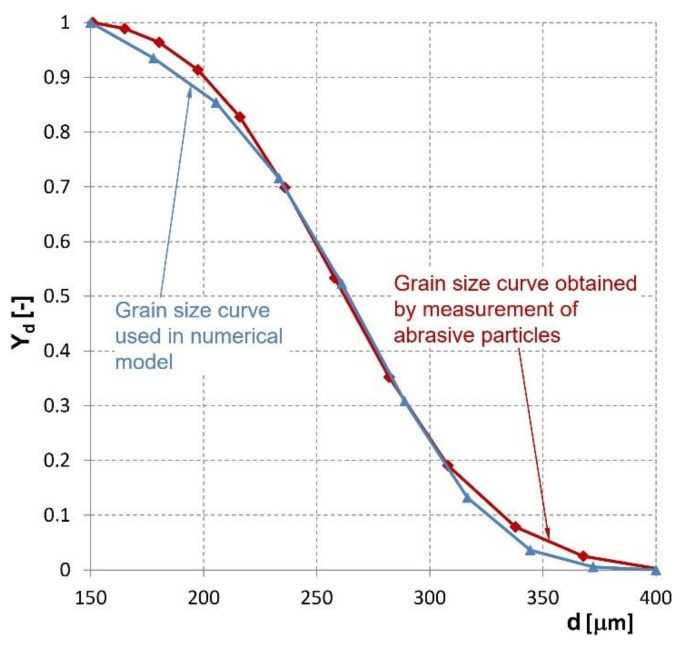
Comparison of the abrasive grain size curves obtained by measurement and the grain size curve used in the numerical model.

**Table 1:** Results of the measured feeding water pressures, volume flow rates, and power distribution in the water nozzle.

p_avg_ (MPa)	SD (MPa)	SEM (MPa)	95% CI (MPa)	Q_avg_ (L/min)	SD (L/min)	SEM (L/min)	95% CI (L/min)	P_avg_ (kW)	SD (kW)	SEM (kW)	95% CI (kW)
105.56	1.68	0.22	105.98 105.14	1.58	0.01	0.001	1.56 1.57	2.77	0.05	0.006	2.79 2.76
194.46	15.27	1.95	198.29 190.64	2.08	0.08	0.010	2.10 2.06	6.75	0.75	0.095	6.94 6.56
302.72	2.13	0.27	303.26 302.19	2.53	0.02	0.001	2.53 2.52	12.76	0.16	0.019	12.80 12.72
406.17	3.22	0.41	406.98 405.36	2.86	0.02	0.002	2.75 2.74	19.36	0.17	0.002	19.41 19.32

p_avg_: average value of the water pressure; Q_avg_: average value of the volume flow rate; P_avg_: average value of the power; SD: standard deviation; SEM: standard error of the mean; 95% CI: 95% confidence interval of values: upper limit and lower limit.

**Table 2:** Comparison of the average values of abrasive particle velocities from CFD calculations and PTV measurement for an abrasive mass flow rate of 100 g/min.

CFD Calculations						PTV Measurement				
*p* (MPa)	N	v_avg_ (m/s)	SD (m/s)	SEM (m/s)	95%CI (m/s)	N	v_avg_ (m/s)	SD (m/s)	SEM (m/s)	95%CI (m/s)
105.56	3059	296.07	113.79	2.05	300.10 292.04	1565	329.34	59.45	1.50	332.28 326.39
194.46	3060	400.09	159.71	2.89	405.75 394.42	2650	455.36	69.62	1.35	458.01 452.70
302.72	3054	493.39	205.39	3.72	500.76 486.19	1489	579.71	75.85	1.97	583.57 575.86
406.17	3056	566.06	242.27	4.38	574.65 557.47	2421	690.38	70.55	1.43	693.20 687.57

*p*: feeding water pressure level; N: number of abrasive particles; v_avg_: average velocity of abrasive particles; SD: standard deviation; SEM: standard error of the mean; 95%CI: 95% confidence interval of values: upper limit and lower limit.

**Table 3:** Comparison of the average values of abrasive particle velocities from CFD calculations and PTV measurement for an abrasive mass flow rate of 200 g/min.

CFD Calculations						PTV Measurement				
*p* (MPa)	N	v_avg_ (m/s)	SD (m/s)	SEM (m/s)	95% CI m/s]	N	v_avg_ (m/s)	SD (m/s)	SEM (m/s)	95% CI (m/s)
105.56	3060	285.01	107.33	1.94	288.81 281.21	3329	281.19	66.51	1.15	283.45 278.94
194.46	3058	388.61	153.81	2.78	394.07 383.16	3742	395.23	85.72	1.40	397.48 392.48
302.72	3059	488.95	193.68	3.50	495.81 565.62	3318	528.87	76.43	1.33	531.48 526.28
406.17	3059	557.36	233.34	4.22	565.63 549.07	4830	641.75	76.65	1.10	643.91 639.58

*p*: feeding water pressure level; N: number of abrasive particles; v_avg_: average velocity of abrasive particles; SD: standard deviation; SEM: standard error of the mean; 95% CI: 95% confidence interval of values: upper limit and lower limit.

**Table 4:** Comparison of the average values of abrasive particle velocities from CFD calculations and PTV measurement for an abrasive mass flow rate of 300 g/min.

CFD Calculations						PTV Measurement				
*p* (MPa)	N	v_avg_ (m/s)	SD (m/s)	SEM (m/s)	95% CI (m/s)	N	v_avg_ (m/s)	SD (m/s)	SEM (m/s)	95% CI (m/s)
105.56	3055	276.86	99.28	1.80	280.38 273.34	6483	251.81	63.55	0.79	253.36 250.27
194.46	3057	379.18	146.07	2.64	384.36 374.00	3350	365.40	82.14	1.42	368.18 362.62
302.72	3059	475.18	188.74	3.41	482.27 468.89	2218	479.52	86.59	1.84	483.13 475.92
406.17	3055	553.22	200.29	3.99	561.03 545.41	6372	601.70	80.28	1.01	603.68 599.73

*p*: feeding water pressure level; N: number of abrasive particles; v_avg_: average velocity of abrasive particles; SD: standard deviation; SEM: standard error of the mean; 95% CI: 95% confidence interval of values: upper limit and lower limit.

**Table 5:** Comparison of the average values of abrasive particle velocities from CFD calculations and PTV measurement—abrasive mass flow rate of 400 g/min.

CFD Calculations						PTV Measurement				
*p* (MPa)	N	V_avg_ (m/s)	SD (m/s)	SEM (m/s)	95% CI(m/s)	N	V_avg_ (m/s)	SD (m/s)	SEM (m/s)	95% CI (m/s)
105.56	3055	269.31	92.54	1.67	272.59 266.02	5205	237.42	57.46	0.80	238.98 235.86
194.46	3059	375.86	133.85	2.42	380.60 371.12	4655	333.54	83.26	1.22	335.94 331.15
302.72	3056	473.57	174.53	3.15	479.75 467.38	2777	433.15	103.73	1.97	437.01 429.29
406.17	3055	549.16	208.90	3.78	556.57 541.76	6920	562.12	82.40	0.99	564.06 560.18

*p*: feeding water pressure level, N: number of abrasive particles, v_avg_: average velocity of abrasive particles, SD: standard deviation; SEM: standard error of the mean; 95% CI: 95% confidence interval of values: upper limit and lower limit.

## Data Availability

The data presented in this study are available on request from the corresponding author.
